# Plant Growth-Promoting Ability of Mycorrhizal *Fusarium* Strain KB-3 Enhanced by Its IAA Producing Endohyphal Bacterium, *Klebsiella aerogenes*

**DOI:** 10.3389/fmicb.2022.855399

**Published:** 2022-04-15

**Authors:** Sheng Cheng, Jian-Wei Jiang, Li-Tao Tan, Jian-Xin Deng, Peng-Yu Liang, Hang Su, Zheng-Xiang Sun, Yi Zhou

**Affiliations:** Department of Plant Protection, College of Agriculture, Yangtze University, Jingzhou, China

**Keywords:** endohyphal bacterium, mycorrhizal *Fusarium*, *Klebsiella aerogenes*, IAA, plant growth promoting

## Abstract

*Fusarium oxysporum* KB-3 had been reported as a mycorrhizal fungus of *Bletilla striata*, which can promote the seed germination and vegetative growth. Endohyphal bacteria were demonstrated in the hyphae of the KB-3 by 16S rDNA PCR amplification and SYTO-9 fluorescent nucleic acid staining. A strain *Klebsiella aerogenes* KE-1 was isolated and identified based on the multilocus sequence analysis. The endohyphal bacterium was successfully removed from the wild strain KB-3 (KB-3^−^), and GFP-labeled KE-1 was also transferred to the cured strain KB-3^−^ (KB-3^+^). The production of indole-3-acetic acid (IAA) in the culturing broths of strains of KE-1, KB-3, KB-3^−^, and KB-3^+^ was examined by HPLC. Their IAA productions were estimated using Salkowski colorimetric technique. The highest concentrations of IAA were 76.9 (at 48 h after inoculation), 31.4, 9.6, and 19.4 μg/ml (at 60 h after inoculation), respectively. Similarly, the three fungal cultural broths exhibited plant promoting abilities on the tomato root and stem growth. The results indicated that the ability of mycorrhizal *Fusarium* strain KB-3 to promote plant growth was enhanced because its endohyphal bacterium, *Klebsiella aerogenes* KE-1, produced a certain amount of IAA.

## Introduction

Endohyphal bacterium (EHB), also called fungal endobacterium or endofungal bacterium, inhabits in fungal hyphae. It is very important to exploit the effects of their species, growth, development, distribution, and secondary metabolites on the host which are little known (Arora and Riyaz-Ul-Hassan, 2019). Bacteria living in fungal cells were first found in the *Endogone* spores (Mosse, 1970), called Bacteria-like organisms. It is later described as EHB (Varma et al., 1981; Tilak et al., 1989). Subsequently, many publications have demonstrated that EHBs are ubiquitous in a variety of fungi, including Ascomycetes (Hoffman and Arnold, 2010; Arendt et al., 2016; Shaffer et al., 2016), Basidiomycetes (Bertaux et al., 2003; Ruiz-Herrera et al., 2015; Glaeser et al., 2016), Mucoromycota (Partida-Martinez, 2017; Desiro et al., 2018), and Glomeromycota (Kluge, 2002; Naumann et al., 2010; Desiro et al., 2013; Agnolucci et al., 2015), which are symbiotic, endophytic, saprotrophic, or pathogenic fungi related to plant (Ibrahim et al., 2008; Araldi-Brondolo et al., 2017; Deveau et al., 2018).

Endohyphal bacterium can provide a great deal of activities to affect the host fungi (Arora and Riyaz-Ul-Hassan, 2019). It can promote the utilization of carbon source (Shaffer et al., 2017) and organic substrates (Guo et al., 2018). It also can affect the sporulation of fungal host (Partida-Martinez et al., 2007b; Lackner et al., 2011; Obasa et al., 2019). For instance, *Rhizopus microsporus* absent its EHB (*Burkholderia rhizoxinica*) loses the capacity of vegetative reproduction, but the sporangium and spore formation are recovered after reintroducing *B. rhizoxinica* (Partida-Martinez et al., 2007b; Lackner et al., 2011). In addition, EHB can promote its fungal host producing bioactive compounds (Partida-Martinez et al., 2007a; Hoffman et al., 2013; Pakvaz et al., 2016; Obasa et al., 2017). For example, *Enterobacter* sp. (EHB) promotes the virulence of *Rhizoctonia solani* by increasing the production of phenylacetic acid (Obasa et al., 2017).

These EHBs tend to play an important role in the interaction between fungi and the plant. Endophytic fungi harboring EHBs are capable of influencing plant endophytic fungal ecology and diversity (Hoffman and Arnold, 2010). Some EHBs can assistant the host endophytic fungi against its competitors and protect the plant *Mediterranean cypress* from invading harmful microorganisms (Pakvaz et al., 2016). *Luteibacter* sp. enhances the activity of plant cell wall-degrading enzymes secreted by endophytic *Pestalotiopsis neglecta*, which contributes to establishing symbiotic relationship between endophyte and plant (Anca et al., 2009; Arendt, 2015). EHBs help the stability of mycorrhizal fungus in plant and the nutrient assimilation of mycorrhiza which simultaneously help its host plant. *Candidatus* Glomeribacter gigasporarum can increase the fitness of a mycorrhizal fungus (Salvioli et al., 2016). *Burkholderia* sp. and *Paenibacillus polymyxa* promote the absorption and transport of phosphate in *Gigaspora margarita*—an arbuscular mycorrhizal fungus (Ruizlozano and Bonfante, 1999; Cruz et al., 2008).

The plant hormone indole-3-acetic acid (IAA) controls the differentiation and elongation of roots and the formation of lateral roots (Ryu and Patten, 2008). It also has been reported to promote the formation of root hairs and various important physiological functions in plants (Vacheron et al., 2013). IAA can be synthesized by a variety of plant-related microorganisms, which includes fungi and bacteria (Duca et al., 2014). A mycorrhizal fungus, *Fusarium oxysporum* KB-3 colonized in root cortex cells of *Bletilla striata*, was reported to significantly promote vegetative growth and seed germination of *B. striata* (Jiang et al., 2019). However, the mechanism is not clear yet. In the study, endohyphal bacteria were detected in the hyphae of *F. oxysporum* KB-3 by 16S RNA PCR amplification and SYTO-9 fluorescent nucleic acid staining approaches. To find the correlation between the EHB and *F. oxysporum* KB-3, EHB was isolated and identify based on sequence analysis. The IAA production and the plant promoting growth abilities were determined among wild strain KB-3, cured strain KB-3 (KB-3^−^), restored strain KB-3 (KB-3^+^), and the EHB.

## Materials and Methods

### Detection of EHB in *Fusarium oxysporum* KB-3

*Fusarium oxysporum* KB-3 as a mycorrhizal fungus of *Bletilla striata* (Jiang et al., 2019) was used for the detection of EHB. It was inoculated on potato dextrose agar (PDA) and cultured at 28°C for 5 days to collect the mycelia. The genomic DNA was extracted by CTAB method (Stenglein and Balatti, 2006). To detect endohyphal bacterial DNA in the fungal culture, 16S ribosomal DNA (16S rDNA) region was used for PCR amplification with primer pair of ER10 (5′-GGCGGACGGGTGAGTAA-3′) and ER11 (5′-ACTGCTGCCTCCCGTAG-3′) (Widjojoatmodjo et al., 1994). The PCR consisted of 2 μl gDNA, 10 μm forward primer, 10 μm reverse primer, 2 × PCR Master Mix (Takara, Japan). It was programmed on GeneAmpR PCR System 2700 (Applied Biosystems, USA) under the following condition: 94°C for 2 min, 35 cycles of denaturation at 94°C for 30 s, annealing at 55°C for 15 s, extension at 72°C for 30 s, and final extension at 72°C for 10 min. To further determine the existence of bacteria in the mycelium, fungal hyphae stained with SYTO-9 green fluorescent nucleic acid solution (Obasa et al., 2017) were observed under a fluorescence Nikon ECLIPSE Ni-U microscope system (Nikon, Japan). The hyphae were collected from the edge of 3-day-old PDA colony, stained with 500 μl SYTO-9 solution (10 μL SYTO-9 with 490 μl Mcilvaine buffer) for 2 min, and rinsed three times with 1 ml Mcilvaine buffer for observation.

### Isolation of EHB

To isolate EHB from *Fusarium oxysporum* KB-3, the method was referred to the study of Obasa et al. (2017). The fungus was inoculated in potato dextrose broth (PDB) shaking incubated with 200 rpm at 28°C for 72 h. The hyphae were collected, disinfected with 70% ethanol for 2 min, and washed with sterile distilled water for 5 times. The fifth washing liquid was spread on Luria-Bertani (LB) agar medium grown at 30°C for 48 h to examine possible bacterial contamination. Meanwhile, the remaining fifth solution was centrifuged at 12,000 g for 10 min to concentrate the possible bacteria for the PCR amplification of 16S rDNA (primers: ER10 and ER11) region as previously mentioned. The surface sterilized hyphae were transferred to a sterile mortar supplemented with silica sand and distilled sterile water and grounded thoroughly with a sterile stick. The resulted solution was grown on LB agar at 28°C to obtain bacterial colonies. The upcoming colonies were further purified and deposited in glycerol solution at −80°C.

### Identification of EHB

The genomic DNA of obtained bacterium was extracted according the procedure of E.Z.N.A.^®^ Bacterial DNA Kit (Omega BIO-TEK, USA). A number of three gene regions of 16S rDNA (Galkiewicz and Kellogg, 2008), DNA gyrase subunit B (*gyrB*) (Deletoile et al., 2009), and RNA polymerase subunit B (*rpoB*) (Deletoile et al., 2009) were amplified to identify the EHB using primer pairs of 27F/1492R, gyrB3/gyrB4, and Vic3/Vic2, respectively. The PCR amplification was conducted in a 40 μl mixture using the regents as previously reported under the referenced conditions (Galkiewicz and Kellogg, 2008; Deletoile et al., 2009). Successful products were purified and sequenced by BGI (Wuhan, China) using two specific primers for each gene region. The resulting sequences were determined by BLAST searches in GenBank and also deposited with accession numbers. The three gene sequences of the present bacterium and its relevant species were edited manually and concatenated in MEGA v.7.0.26 (Kumar et al., 2016). A phylogenetic analysis was conducted by Bayesian inference (BI) using MrBayes v.3.2.1 (Ronquist et al., 2012) based on a Markov Chain Monte Carlo (MCMC) algorithm. The best-fit nucleotide substitution model was specified by jModelTest 2.1.7 (Darriba et al., 2012) under default settings followed by Akaike information criterion (AIC). Trees were sampled every 100 generations from 5,000,000 generations resulting in total 50,000 trees. The first 12,500 trees (first 25% samples by default in the software) were discarded representing the burn-in phase and the remaining 37,500 trees were for calculating posterior probability (PP) values in the majority rule consensus tree.

### Preparation of Cured *Fusarium oxysporum* KB-3 (KB-3^–^)

The wild strain of *Fusarium oxysporum* KB-3 was inoculated in PDB medium containing 50 μg/ml tetracycline, 70 μg/ml gentamicin, 0.1 M CaCl_2_, and 1% DMSO, incubated at 28°C for 72 h. Every three times sub-cultured fungus was determined by PCR amplification of 16S rDNA region (primers: ER10 and ER11) to detect the EHB. If the 16S rDNA amplicon was still present, antibiotic treatment would be continued until it disappeared. In the meantime, the resulted bacteria-free hyphae were stained with SYTO-9 solution and examined under the fluorescence microscope as previously mentioned to examine the bacterial existence. The successful cured strain (KB-3^−^) was confirmed by both methods and deposited in glycerol solution preserved at −80°C for future use.

### Symbiotic Reconstruction of Restored *Fusarium oxysporum* KB-3 (KB-3^+^)

To visualize *Klebsiella aerogenes* KE-1 in the strain KB-3, the bacterium was labeled with green fluorescent protein (GFP) and restored to the cured strain KB-3^−^. First, the bacterium was transformed by electroporation with plasmid pLac-EGFP-Chl-signal-Hyg. In the meantime, the protoplasts of cured strain KB-3^−^ were prepared as previously described by Lakshman et al. (2012). Subsequently, the GFP-labeled bacteria and protoplasts were separately rinsed with precooled shock buffer (0.6 mol/L mannitol, 0.1 g/L CaCl_2_) for three times, and then, both were mixed and placed in ice bath for 10 min. The mixture was converted by electroporation at 200 Ω, 25 μF in 2-mm cuvettes (voltage range up to 5 kV cm^−1^). The mixture (100 μl) was inoculated in 0.5 ml 1/4 PDB medium incubated for 12 h at 28°C. Afterward, the mixture was spread on 1/4 PDA and then covered a layer of 1/4 PDA medium to form a double-layer plate. Once hyphae grew through the upper medium, the hyphal tip was transferred to new medium. It was repeated for several times to ensure no bacteria attached the hyphae. The hyphae were observed under the fluorescence microscope as previously mentioned. The successfully restored strains (KB-3^+^) were deposited and also determined by PCR amplification of 16S rDNA using primers of ER10 and ER11.

### IAA Examination

To detect IAA production from the cultural broth of *Klebsiella aerogenes* KE-1, wild strain KB-3, cured strain KB-3^−^ and restored strain KB-3^+^, it was examined using high-performance liquid chromatography (HPLC) compared with IAA standard. A number of three mycelium blocks (9 mm in diam.) for fungal strain grown on PDA for 5 days were transferred into 200 ml LB liquid comprising 10 mg/ml L-tryptophan, cultured at 200 rpm and 28°C. Meanwhile, 100 μl LB cultural solution of the bacterium KE-1 (OD_600_ = 0.6) was inoculated as previous treatment for each fungal strain. After incubated for 60 h, each filtrate (100 ml) collected by filtration with lens wiping paper and centrifugation was extracted twice with 100 ml ethyl acetate (EtOAc). The EtOAc layers were condensed to dry by rotary evaporation vacuum (45°C). The residues were dissolved in methanol to 10 ml and filtered by a 0.22-μm Millipore filter (Merck Millipore, Germany) for HPLC analysis. IAA (Sigma, USA) was dissolved in methanol as a standard sample. Besides, ten-time-diluted KE-1 extraction was used for HPLC analysis. Venusil XBP-C18 column (4.6 × 250 mm, 5 μm) was used to analyze the EtOAc extract in Wooking HPLC system (Wooking K2025, China). The mobile phase was acetonitrile and 2% acetic acid aqueous solution at the flow rate of 1 ml/min, and the injection volume was 10 μl. The results were analyzed by Wookinglab software v00.02.20.00 (Wooking, China).

### IAA Evaluation

To evaluate the IAA production of *Klebsiella aerogenes* KE-1, wild strain KB-3, cured strain KB-3^−^, and restored strain KB-3^+^, it was conducted using Salkowski colorimetric technique (Ehmann, 1977; Glickmann and Dessaux, 1995; Huddedar et al., 2002; Chaiharn and Lumyong, 2011). After every 12 h of incubation using previous culturing method for IAA examination (3 replications), 2 ml culture broth (*n* = 3) was taken and centrifuged, followed by 1 ml supernatant treated with 2 ml of Salkowski reagent (1 m, 0.5 mm FeCl_3_, and 50 ml, 35% HClO_4_) (Chaiharn and Lumyong, 2011). The samples were incubated at 25°C for 30 min in the dark and evaluated optical density (OD) values at 530 nm by spectrophotometer (BIO-RAD, USA). LB medium containing 10 mg/ml L-tryptophan was used as control. The experiment was repeated for two times. IAA concentration was calculated using IAA standard curve (y = 0.0238; x = 0.0395) determined by different concentrations IAA (Sigma, USA) solutions.

### Plant Growth Promoting Assay

The LB containing 10 mg/ml L-tryptophan without NaCl cultural supernatants of wild strain KB-3, cured strain KB-3^−^, and restored strain KB-3^+^ was prepared as previous treatment for IAA examination. After incubated for 60 h, each supernatant was modified to pH 7.0 ± 0.1 with 0.5 M NaOH if needed and filtered two times by the 0.22-μm Millipore filter (Merck Millipore, Germany) to obtain the 20-ml filtrate. The filtrates were used for seedling growth promotion assay. Tomato seeds were surface-disinfected with 75% ethanol for 2 min and 4% sodium hypochlorite for 5 min and then rinsed with distilled sterile water for 5 times. The surface sterilized seeds were transferred to 90 mm Petri dishes with moistened sterile filter paper to accelerate seed germination. Healthy similar sprouted seeds (*n* = 10) were randomly transferred to crisper containing filter papers saturated with distilled water (5 ml). Per seedling was covered with 50 μl each of previous LB cultural supernatants and LB liquid containing 10 mg/ml L-tryptophan without NaCl (control) (Hoffman et al., 2013). Each treatment had three replicates (i.e., 30 seedlings/treatment). The crispers were tilted placing in an illumination incubator at 25°C for 5 days (12-h light/dark period). Stem and root lengths of tomato seedlings were measured and the experiment was repeated for two times.

### Statistical Analysis

Data obtained from the IAA production and plant growth-promoting assay were subjected to the analysis of variance (ANOVA) with SPSS 17.0 software (SPSS Inc., Chicago, IL, USA). The results were presented as average means and standard deviation. Duncan's multiple range test was used to compare the mean value at the 5% (*p* < 0.05) level of significance.

## Results

### Existence of EHB of *Fusarium oxysporum* KB-3

**Figure 1:**
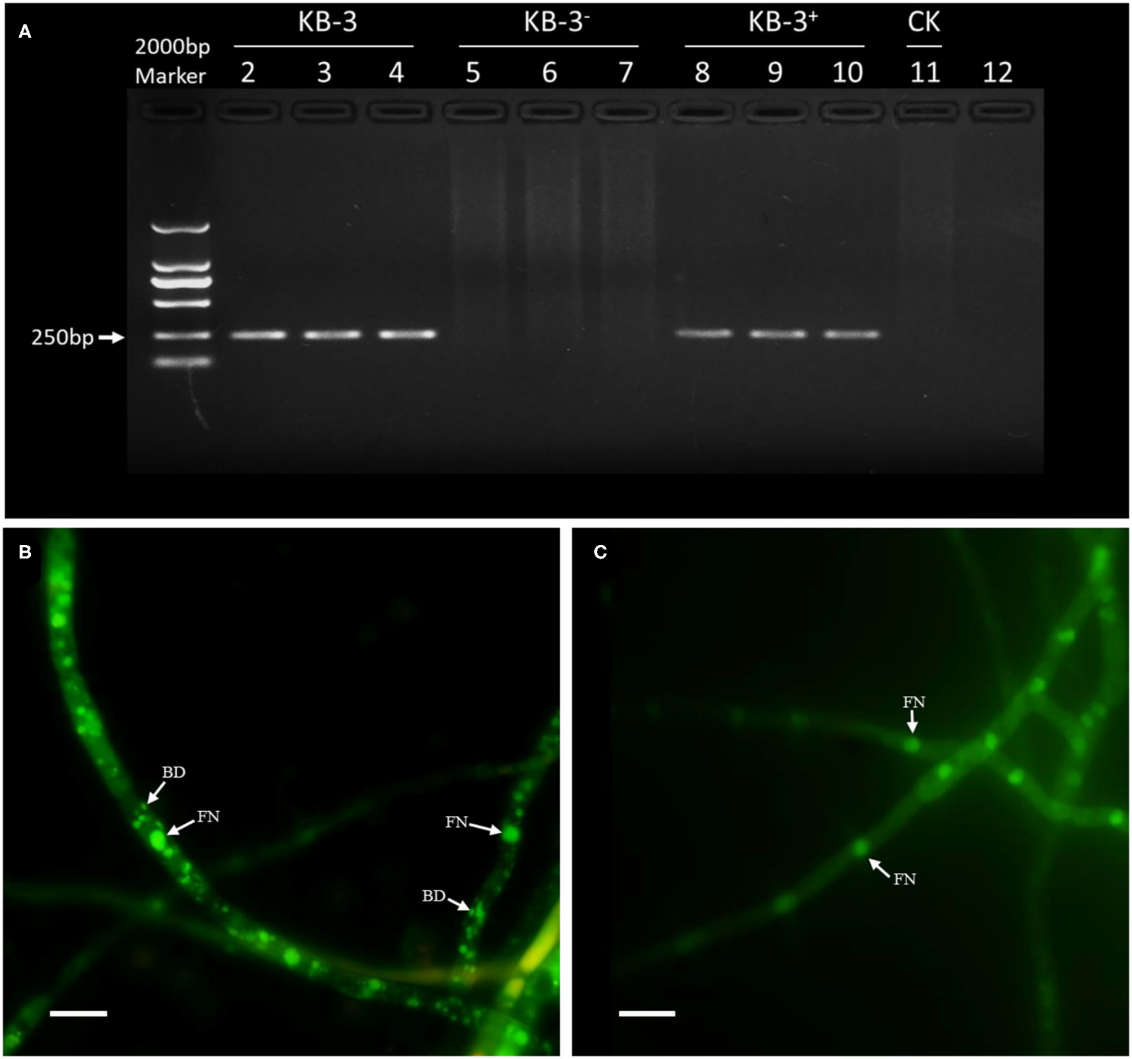
The presence of endohyphal bacteria of *Fusarium oxysporum* KB-3. **(A)** PCR amplification of 16S rDNA for wild strain KB-3 (KB-3, lanes 2–4), cured strain KB-3 (KB-3^−^, lanes 5–7), restored strain KB-3 (KB-3^+^, lanes 8–10), and the final rinse solution of wild strain for endohyphal bacterial isolation (lane 11). **(B)** SYTO-9 fluorescent nucleic acid stained the wild strain KB-3 hyphae. **(C)** SYTO-9 fluorescent nucleic acid stained the cured strain KB-3^−^ hyphae. FN, fungal nucleus; BD, bacterial DNA. Bars = 10 μm.

The PCR amplification of 16S rDNA for either the wild strain KB-3 and restored strain KB-3^+^ resulted a band of 255-bp fragment; however, the cured strain KB-3^−^ and the final rinse solution of wild strain for endohyphal bacterial isolation remained empty (Figure 1). The resulted showed that the cured strain KB-3^−^ was successfully obtained by continuously cultured in modified PDB. Similarly, hyphae of the wild strain KB-3 (Figure 1B) contained the fungal nucleus and also bacterial DNA under fluorescence microscopy, while the cured strain KB-3^−^ (Figure 1C) only comprised fungal nucleus.

### Isolation and Identification of EHB

**Figure 2:**
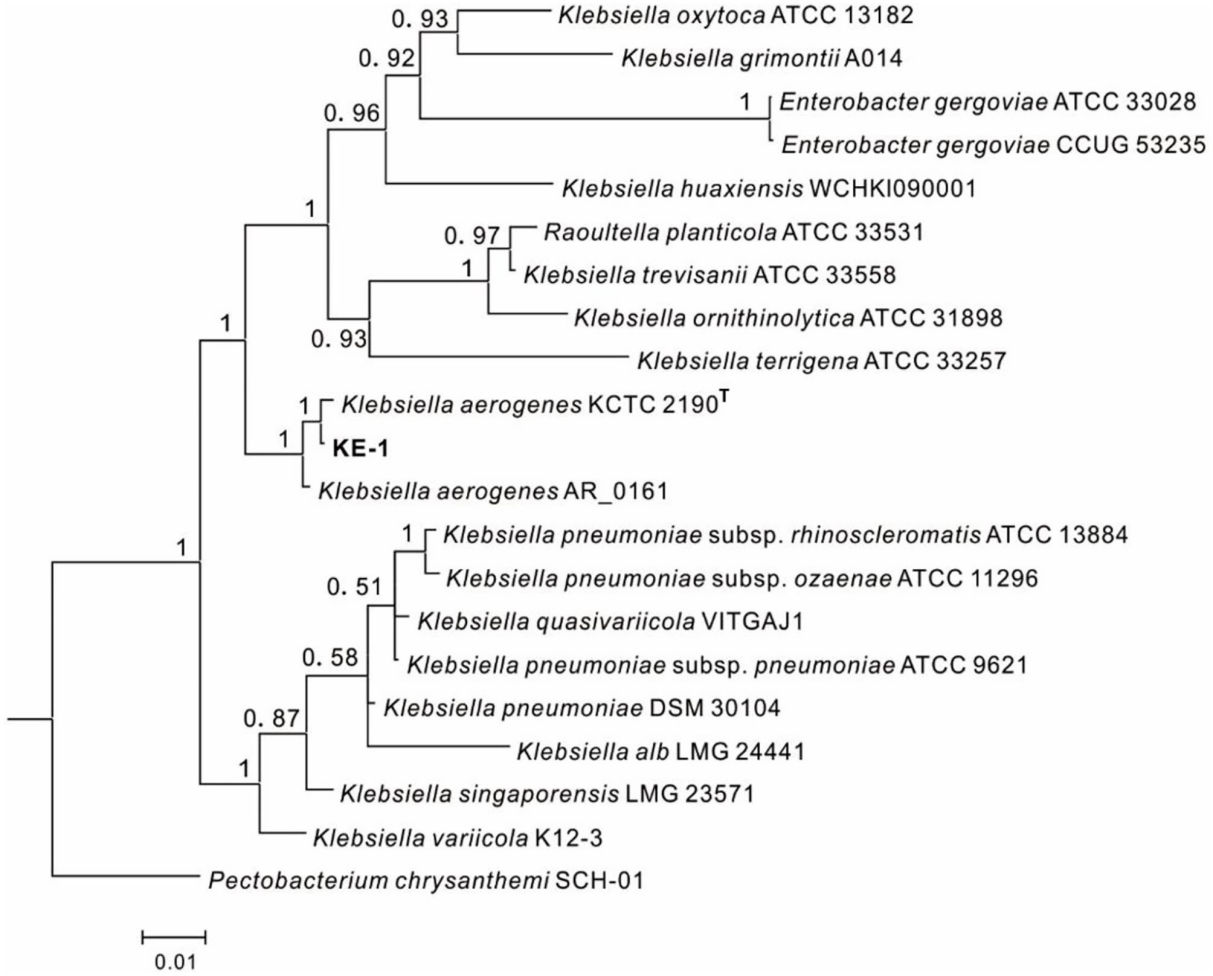
Bayesian tree of endohyphal bacterium *Klebsiella aerogenes* KE-1 on the basis of three gene (16S rDNA, *gyrB*, and *rpoB*) sequences. Bayesian posterior probabilities >0.5 are given at the nodes. Examined strains are in bold. The out group is *Pectobacterium chrysanthemi* SCH-01.

One EHB (KE-1) was obtained from surface disinfected and grounded hyphae of *Fusarium oxysporum* KB-3 on LB agar. The fifth washing liquid obtained from hyphal surface sterilization was no bacterial colonies observed on LB agar plates, which came out that the hyphae were completely disinfected. To identify the bacterium KE-1, the 16S rDNA (1,440 bp), *gyrB* (506 bp), and *rpoB* (512 bp) gene sequences were amplified and deposited in GenBank database with accession numbers of MZ144165, MZ150560, and MZ150561, respectively. BLAST searches showed that each of the three gene sequences shared 99–100% identity with that of *Klebsiella aerogenes* (Syn. *Enterobacter aerogenes*) strains, for example, the type strain KCTC 2190 (Taxonomy ID: 1028307). Bayesian tree (Figure 2) based on the combined 16S rDNA, *gyrB* and *rpoB* gene sequence dataset was constructed with *Klebsiella* spp.. The results clearly showed that the EHB KE-1 was *K. aerogenes* and it was clustered together with the type strain KCTC 2190 and *K. aerogenes* AR_0161 (NCBI genome accessions CP028951) supported with high PP values (1.0), from which the present bacterium formed a sub-clade with strain KCTC 2190 (PP = 1.0).

### Cured (KB-3^–^) and Restored (KB-3^+^) *Fusarium oxysporum* KB-3

**Figure 3:**
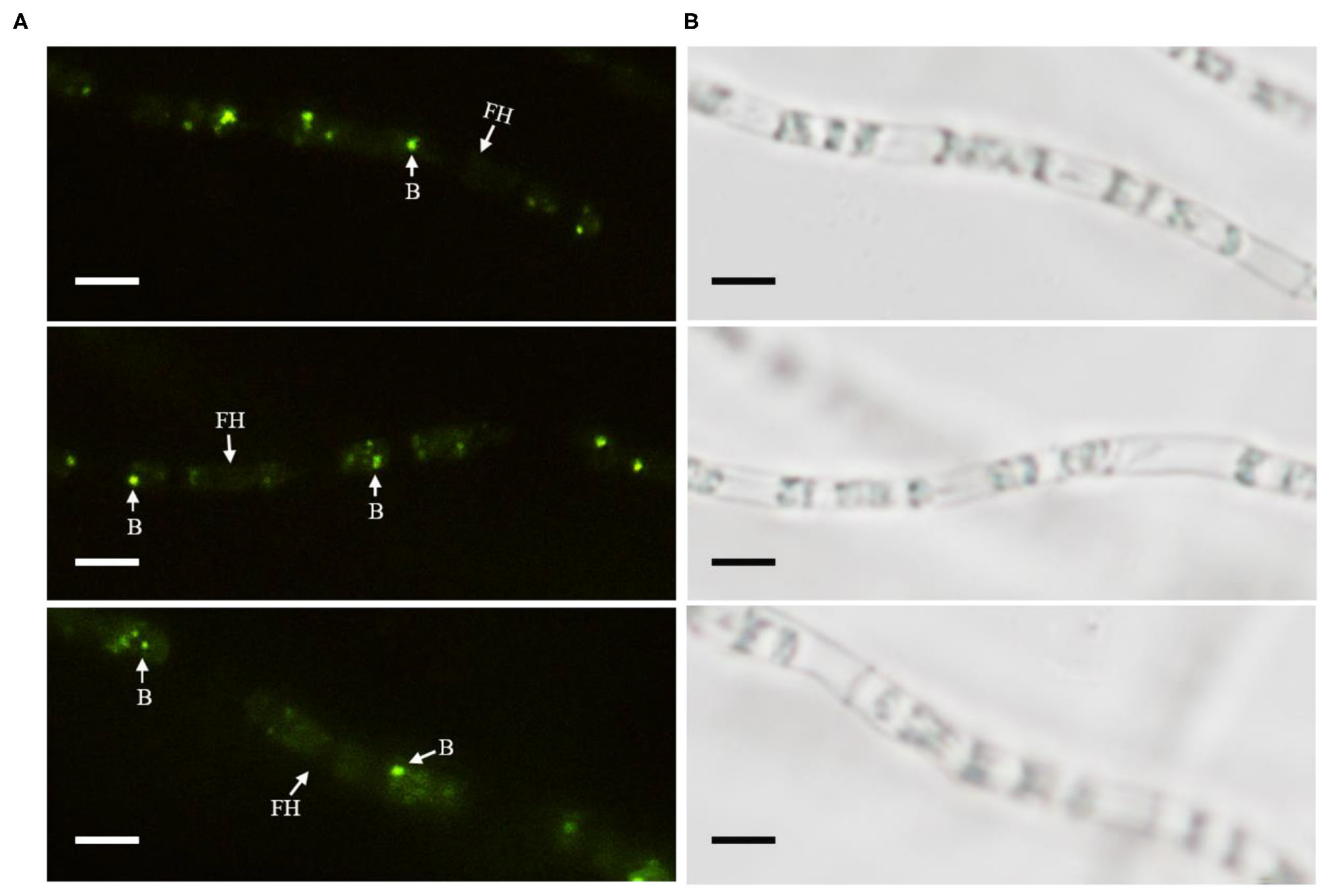
GFP-labeled *Klebsiella aerogenes* localized in *Fusarium oxysporum* KB-3 hyphae. **(A)** Hyphae under fluorescence microscopy (left) **(B)** the same one under white light (right). Bars = 10 μm.

The cured strain KB-3^−^ was gained after continuously culturing wild *Fusarium oxysporum* KB-3 in modified PDB broth (Figures 1A,C). Meanwhile, the GFP-labeled *Klebsiella aerogenes* KE-1 was obtained by the electroporation and it was also successfully restored in the hyphae of the cured strain KB-3^−^ (KB-3^+^) (Figure 1A) observed by fluorescence microscopy (Figure 3). The GFP-labeled *K. aerogenes* KE-1 bacteria could be precisely exhibited in the hyphae of the restored strain KB-3^+^.

### IAA Examination and Evaluation

**Figure 4:**
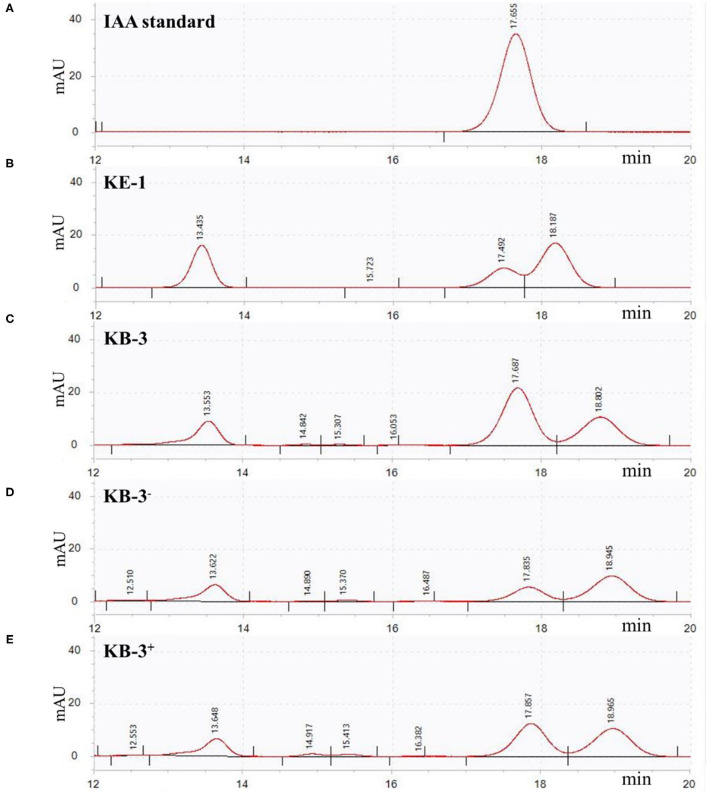
HPLC results for **(A)** IAA standard, and filtrate extracts from **(B)** endohyphal bacterium *Klebsiella aerogenes* KE-1, **(C)** wild strain KB-3 of *Fusarium oxysporum* (KB-3), **(D)** cured strain KB-3 (KB-3^−^) and **(E)** restored strain KB-3 (KB-3^+^) at 60 h of inoculation in the Luria-Bertani (LB) containing 10 mg/ml L-tryptophan. Elution time was 17.65 min for the IAA standard, 17.49 for KE-1, 17.68 for wild strain KB-3, 17.83 for cured strain KB-3 (KB-3^−^), and 17.85 for restored strain KB-3 (KB-3^+^). The small difference in retention time reflected partial masking of constituents in the sample matrix, a common phenomenon for HPLC analysis using reversed-phase columns.

**Figure 5:**
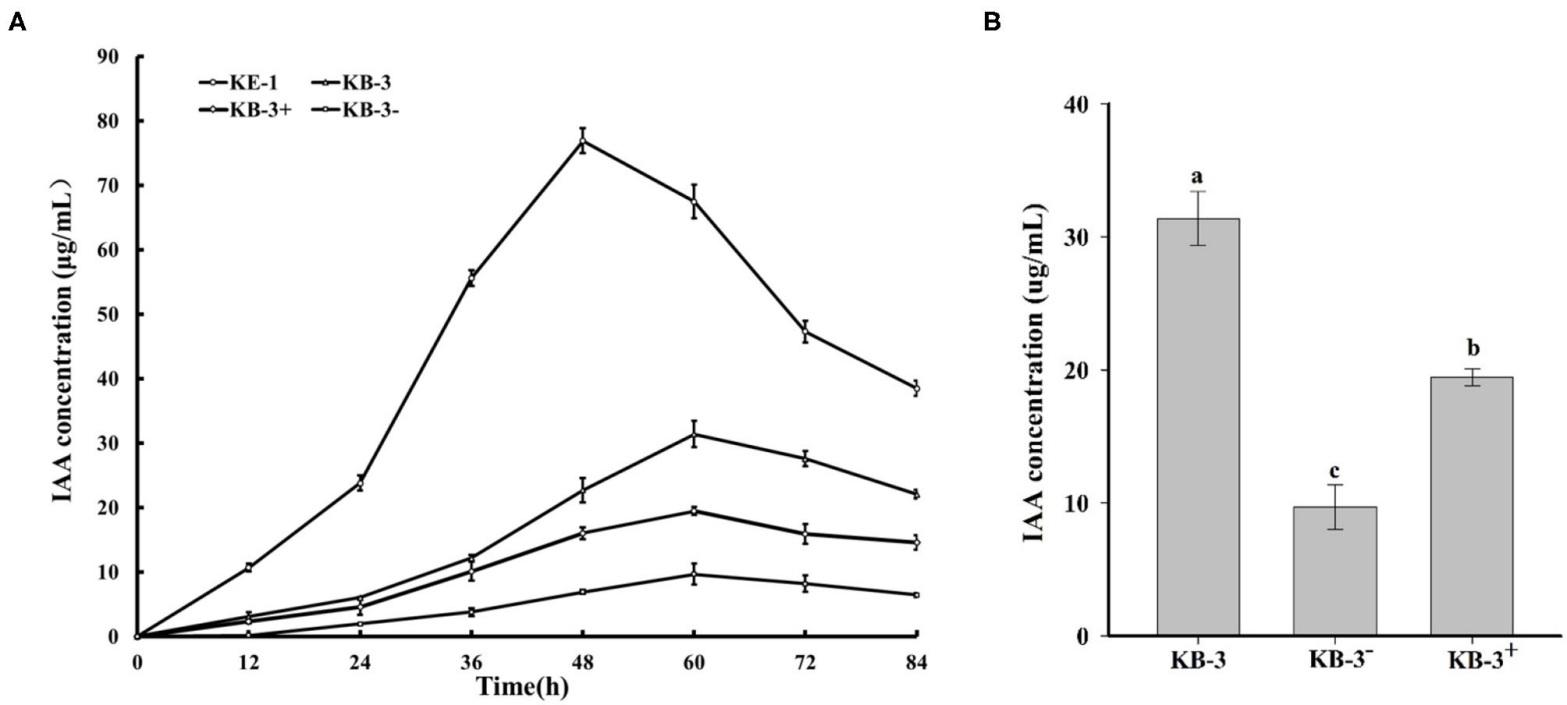
**(A)** IAA production in the Luria-Bertani (LB) containing 10 mg/ml L-tryptophan cultural broth of wild strain KB-3 of *Fusarium oxysporum* (KB-3), cured strain KB-3 (KB-3^−^), restored strain KB-3 (KB-3^+^) and its endohyphal bacterium *Klebsiella aerogenes* KE-1 (KE-1) during 84 h post-inoculation. **(B)** IAA production in the Luria-Bertani (LB) containing 10 mg/ml L-tryptophan cultural broth of wild strain KB-3 of *Fusarium oxysporum* (KB-3), cured strain KB-3 (KB-3^−^), restored strain KB-3 (KB-3^+^) at 60 h after inoculation. The points in **(A)** and columns in **(B)** represented the means of two separate experiments, each with three replicates. The interval bars represented the standard deviation of the means. Different letters marked on the top of columns were significantly different according to Duncan's multiple range test at *p* < 0.05.

In HPLC analysis, IAA production was found in the cultural broth of *Klebsiella aerogenes* KE-1, wild strain KB-3, cured strain KB-3^−^, and restored strain KB-3^+^ (Figure 4). It was evaluated every 12 h by the Salkowski colorimetric technique within 84 h of inoculation. The IAA production of all the tested samples was gradually increased and then decreased after the highest (Figure 5). However, the EHB could secrete the highest amount of IAA (76.9 μg/ml) after 48 h inoculation (Figure 5A) and the three fungal strains secreted the highest IAA production at 60 h post-inoculation. At the highest IAA production, the wild stain KB-3 produced the highest amount of IAA (av. 31.4 μg/ml), followed by the restored strain KB-3^+^ (av. 19.4 μg/ml), and then the cured strain KB-3^−^ (av. 9.6 μg/ml) (Figure 5B). The IAA production was significantly different (*p* < 0.05) among the three fungal strains. In addition, it was noteworthy that the IAA production of restored strain KB-3^+^ did not reach to the same level of wild strain KB-3 (around three-fifths of the wild one), but significantly higher than the cured strain KB-3^−^ produced.

### Plant Growth Promoting Assay

**Figure 6:**
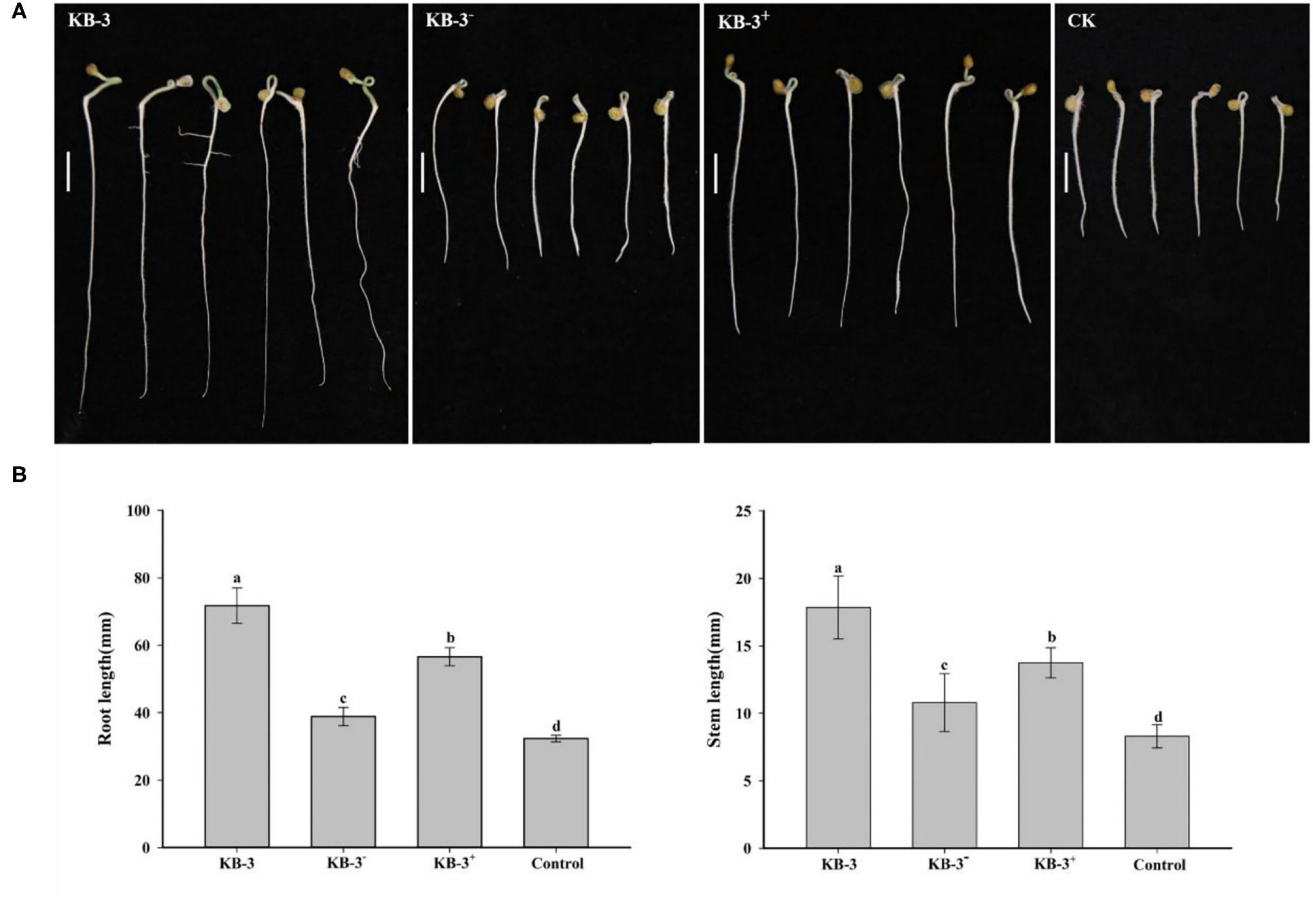
Growth-promoting assays for tomato treated with sterile filtrated Luria-Bertani (LB) containing 10 mg/ml L-tryptophan cultural broth of wild strain KB-3 of *Fusarium oxysporum* (KB-3), cured strain KB-3 (KB-3^−^), restored strain KB-3 (KB-3^+^) and LB broth containing 10 mg/ml L-tryptophan (control) after 5 days. **(A)** The growth situation of tomato seedlings under different treatments. Bars = 10 mm. **(B)** The means (*n* = 30) root (left) and stem (right) lengths of tomato seedlings. The interval bars represented the standard deviation of the means. Different letters marked on the top of columns were significantly different according to Duncan's multiple range test at *p* < 0.05.

The wild *Fusarium oxysporum* strain KB-3, its cured strain KB-3^−^, and its restored strain KB-3^+^ exhibited plant growth-promoting effects on tomato using their fermentation broth compared with the controls (Figure 6). The results were consistent with their IAA production abilities. The root length and stem length of tomato seedlings treated with the wild strain KB-3 (around two times of those generated from controls) were the longest, followed by the restored strain KB-3^+^ and then the cured strain KB-3^−^. Similarly, the restored strain KB-3^+^ induced shorter length of both tomato root and stem than those induced by the wild strain but significantly higher than the cured strain KB-3^−^. The root length and stem length of tomato seedlings under the four treatments were significantly different from each other (*p* < 0.05). Besides, lateral root development of tomato seedlings was observed only in the treatment of the wild strain KB-3 (Figure 6).

## Discussion

Previously, we reported that *Fusarium oxysporum* KB-3 was a mycorrhizal fungus of *Bletilla striata* (Jiang et al., 2019). In this study, an EHB strain was isolated and identified as *Klebsiella aerogenes* (Syn. *Enterobacter aerogenes*) from the *Fusarium* strain. It is a Gram-negative bacterium of the family Enterobacteriaceae widely distributed in water, soil, and air (Iyer et al., 2017; Tindall et al., 2017; Mann et al., 2021). It also has been isolated as a plant growth-promoting endophyte or rhizobacterium, an antagonist against plant pathogens, and a bioremediation bacterium to remedy metalliferous pollutants (Rudd et al., 1983; Shang et al., 2019; Harsha and Nair, 2020). Except *K. pneumoniae* reported as an EHB of *Rhizopus oryzae* (Birol and Gunyar, 2021), none species in the genus has been reported as an EHB. *Bradyrhizobium* sp. (Li et al., 2010), *Chitinophaga* sp. (Shaffer et al., 2017), *Enterobacter* sp. (Obasa et al., 2019; Rahayu et al., 2020), *Izhakiella Australiensis* (Rahayu et al., 2020), *Streptococcus* sp., *Curtobacterium* sp., and *Rothia* sp. (Shaffer et al., 2018) have been reported as EHBs from *Fusarium*. To our knowledge, this is the first report of *K. aerogenesis* as an EHB in *F. oxysporum*.

Cured fungal strains in majority studies have been obtained by continuous subculture under an antibiotic condition (Partida-Martinez and Hertweck, 2005; Obasa et al., 2017, 2019). However, it is quite difficult to get the cured strain KB-3^−^. EHB may be protected in some way by the host fungi (Sharma et al., 2008; Guo et al., 2017), and antibiotics cannot be effectively against EHB inside of fungal hyphae. Therefore, CaCl_2_ and DMSO were added to the antibiotic culture medium and successfully obtained the cured strain. The result suggested that the two compounds may alter cell permeability and devote to the effecting of antibiotic. In addition, there are tremendous cases of successful restoring EHB to their host fungi by co-culturing GFP-labeled EHB with mycelium of the host fungi using low-nutrient media, such as 1/4 PDA and M9 medium (Partida-Martinez et al., 2007b; Arendt et al., 2016; Obasa et al., 2017, 2019; Baltrus et al., 2018; Hazarika et al., 2020). Arendt et al. (2016) also reported that EHB colonization occurred more frequently and steadily under nutritional deficiency conditions. However, the restoration of EHB could not be gained by the method in some cases (Guo et al., 2017). Similarly, we did not successfully restore the EHB KE-1 into strain KB-3^−^, even co-cultured with the protoplasts. Finally, it was successfully restored using electrophoresed the EHB and fungal protoplasts mixture and then sub-cultured in low nutrient medium. Those methods may help to the successful implementation of EHB cure and restoration in the future.

*Luteibacter* sp., an EHB from a foliar endophytic fungus of *Pestalotiopsis* aff. *neglecta* could not produce IAA, but it significantly enhanced the IAA production of the endophyte (Hoffman et al., 2013). Similarly, the present EHB *K. aerogenesis* KE-1 increased the IAA production of the host fungus *F. oxysporum* in this study. Differently, it could secrete higher amount of IAA in pure culture. The results indicated that the EHB might execute its IAA producing system to increase the fungal host's IAA producing capability. The view was supported by the previous reports that *Rhizopus microsporus* secrete rhizoxin given by its EHB *Burkholderia* sp. (Partida-Martinez and Hertweck, 2005) and *Ustilago maydis* fixed nitrogen because of its EHB *Bacillus pumilus* (Ruiz-Herrera et al., 2015). Restored *Rhizoctonia Solani* strain (*Enterobacter* sp. EHB) was not completely recovering the phenylacetic acid production (around 3/4) in comparison with the wild one (Obasa et al., 2017). The restored strain KB-3^+^ produced IAA up to three-fifths of that produced by the wild strain in the study. The IAA production decrease may be caused by the less bacterial amount in hyphae of the restored strain compared with the wild strain, or be associated with the existence of other uncultivable EHB of *F. oxysporum* KB-3.

Plant growth promoting is one of the most important roles for symbiotic fungi (Hassan et al., 2013), which can produce various secondary metabolites, including ammonia and plant hormones, especially IAA (Strobel et al., 2004; Fouda et al., 2015). Production of IAA is considered to be a unique feature for promoting plant growth fungi (Muhammad Khan et al., 2010). *F. oxysporum* KB-3 could significantly promote the seed germination and vegetative growth for *B. striata* (Jiang et al., 2019), but also for tomato in this study. The results showed that *K. aerogenesis* KE-1 could enhance the plant growth-promoting ability of its host on tomato, which was consistent with the enhancing of its host IAA production. The cured strain KB-3^−^ showed some abilities to promote plant growth on tomato, which might be associated with producing a certain amount of IAA. Besides, it also might associate with other *Fusarium* metabolites, such as gibberellic acid (Bilal et al., 2018). High percentage of IAA can induce lateral root formation and root hair development (Ludwig-Muller, 2015), which explains that only tomato roots treated with wild strain KB-3 fermentation broth comprise lateral roots. Additionally, fungal IAA has been reported mediating the mutual recognition and relationship establishment between fungi and plants (Mehmood et al., 2018a,b). Hence, *K. aerogenesis* KE-1 might have essential implications for the recognition and colonization between *F. oxysporum* KB-3 and *B. striata*.

## Data Availability Statement

The datasets presented in this study can be found in online repositories. The names of the repository/repositories and accession number(s) can be found at: https://www.ncbi.nlm.nih.gov; MZ144165, MZ150560, and MZ150561.

## Author Contributions

YZ, Z-XS, and SC did the study design. SC and L-TT isolated and identified the EHB and wrote the manuscript. SC, J-WJ, P-YL, and HS contributed to cured and restored strains. J-WJ and P-YL finished experiments the IAA determination and plant growth-promotingassay. J-XD, Z-XS, and YZ were responsible for manuscript revision. YZ and Z-XS led the program and obtained the funding. All authors read and approved the manuscript.

## Funding

This study was financed by the major key project of Hubei Province (2020BBA051).

## Conflict of Interest

The authors declare that the research was conducted in the absence of any commercial or financial relationships that could be construed as a potential conflict of interest.

## Publisher's Note

All claims expressed in this article are solely those of the authors and do not necessarily represent those of their affiliated organizations, or those of the publisher, the editors and the reviewers. Any product that may be evaluated in this article, or claim that may be made by its manufacturer, is not guaranteed or endorsed by the publisher.
